# Beetroot Supplementation as a Nutritional Strategy to Support Post-Exercise Autonomic Recovery in Postmenopausal Women: A Systematic Review and Meta-Analysis

**DOI:** 10.3390/healthcare13192496

**Published:** 2025-10-01

**Authors:** Rodrigo D. Raimundo, Lucas Fornari Laurindo, Fabiana V. M. Gimenez, Jonas Benjamim, Luana A. Gonzaga, Marianne P. C. R. Barbosa, Marina de Morais Martins, Edson H. Ito, Alexandre L. Barroca, Giovanna de J. Brito, Derfel R. M. A. Folegatti, Andrey A. Porto, David M. Garner, Sandra Maria Barbalho, Vitor E. Valenti

**Affiliations:** 1 Laboratório de Delineamento de Estudos e Escrita Científica, Centro Universitário Faculdade de Medicina do ABC, Santo André 09060-650, SP, Brazil; 2 Laboratory for Systematic Investigations of Diseases, Department of Biochemistry and Pharmacology, School of Medicine, Universidade de Marília (UNIMAR), Marília 17525-902, SP, Brazil; lucasffffor@gmail.com (L.F.L.); smbarbalho@gmail.com (S.M.B.); 3 Postgraduate Program in Structural and Functional Interactions in Rehabilitation, School of Medicine, Universidade de Marília (UNIMAR), Marília 17525-902, SP, Brazil; 4 Systematic Reviews Center for Cardiovascular and Metabolic Health, School of Philosophy and Sciences, São Paulo State University (UNESP), Marília 17525-900, SP, Brazil; fabiana.vm.gimenez@unesp.br (F.V.M.G.); luana.gonzaga@unesp.br (L.A.G.); mapenachini@hotmail.com (M.P.C.R.B.); marina.m.martins@unesp.br (M.d.M.M.); itohisayuki@gmail.com (E.H.I.); al.barroca@unesp.br (A.L.B.); giovanna.j.brito@unesp.br (G.d.J.B.); derfel.folegatti@unesp.br (D.R.M.A.F.); aa.porto@unesp.br (A.A.P.); vitor.valenti@unesp.br (V.E.V.); 5 Institute for Physical Activity and Nutrition (IPAN), Deakin University, Burwood, VIC 3125, Australia; j.benjamim@deakin.edu.au; 6 Cardiorespiratory Research Group, School of Biological and Medical Sciences, Faculty of Health and Life Sciences, Oxford Brookes University, Headington Campus, Gipsy Lane, Oxford OX3 0BP, UK; davidmgarner1@gmail.com; 7 Department of Biochemistry and Nutrition, School of Food and Technology of Marília (FATEC), Marília 17500-000, SP, Brazil; 8 UNIMAR Charity Hospital, Universidade de Marília (UNIMAR), Marília 17525-902, SP, Brazil

**Keywords:** beetroot, dietary nitrate, postmenopausal women, heart rate variability, post-exercise recovery

## Abstract

**Background/Objectives**: Beetroot supplementation is a rich source of inorganic nitrate and has been proposed to enhance nitric oxide bioavailability and support cardiovascular recovery after exercise. This study aimed to evaluate the effects of beetroot supplementation on post-exercise cardiovascular and autonomic recovery in postmenopausal women. **Methods**: A systematic review and meta-analysis were conducted according to the PRISMA (Preferred Reporting Items for Systematic Reviews and Meta-Analyses) guidelines. Searches were performed in PubMed, Scopus, and Web of Science databases from inception to July 2025. Ten trials involving postmenopausal women were included. Outcomes assessed included cardiovascular measures (blood pressure and vascular function), autonomic parameters derived from heart rate variability (HRV)—specifically the root mean square of successive differences (RMSSD), the standard deviation of normal-to-normal intervals (SDNN), and high-frequency power (HF)—as well as physical performance (peak oxygen uptake [VO_2peak_ or VO_2max_] and functional fitness tests). Four independent reviewers extracted data, assessed risk of bias, and evaluated the certainty of evidence using the GRADE (Grading of Recommendations, Assessment, Development, and Evaluation) approach. **Results**: Pooled analyses from two trials (*n* = 54) revealed a statistically significant improvement in RMSSD with beetroot supplementation (mean difference: 6.68 ms; 95% CI: 0.86 to 12.50; *p* = 0.02), suggesting enhanced parasympathetic reactivation after exercise. No significant effects were detected for HF (mean difference: 61.75 ms^2^; 95% CI: −70.92 to 194.43; *p* = 0.36) or SDNN (mean difference: 6.20 ms; 95% CI: −9.69 to 22.09; *p* = 0.44). Substantial to considerable heterogeneity was identified across outcomes (I^2^ = 73–86%). Certainty of evidence was rated moderate for RMSSD, low for SDNN, and very low for HF. **Conclusions**: Beetroot supplementation may enhance post-exercise autonomic recovery in postmenopausal women, primarily through improvements in RMSSD. However, further trials with standardized protocols, larger samples, and longer intervention durations are required to clarify its impact on broader HRV domains, cardiovascular function, and clinical outcomes.

## 1. Introduction

Among emerging nutritional strategies, dietary nitrate—abundant in vegetables such as beetroot—has demonstrated promising vascular and autonomic benefits. Once ingested, nitrate (NO^3−^) undergoes entero-salivary circulation and is reduced to nitrite and subsequently to nitric oxide (NO), especially under hypoxic or acidic conditions common during post-exercise recovery [1,2]. As a key signaling molecule, NO promotes vasodilation, regulates blood flow, and enables parasympathetic reactivation—mechanisms particularly relevant to post-exercise cardiovascular recovery [3,4].

Evidence from exercise trials suggests both benefits and limitations of beetroot juice (BJ) supplementation: BJ has been shown to improve blood pressure regulation, endothelial function, and autonomic recovery; however, inconsistent results have been reported when compared to nitrate salts or when used in different exercise modalities [1,5]. Supplementation has also been associated with improvements in skeletal muscle contractile efficiency, oxygen utilization, and physical performance, particularly in endurance contexts. Yet its effectiveness appears to vary in strength-based protocols and among individuals with distinct clinical profiles [6], underscoring the need to clarify the conditions under which BJ supplementation is most beneficial.

Postmenopausal women face a disproportionate risk of cardiovascular dysfunction, largely attributed to reduced estrogen levels that impair nitric oxide synthase (NOS) activity and decrease NO bioavailability [7,8]. This hormonal decline contributes to vascular stiffness, elevated blood pressure, and endothelial dysfunction—recognized markers of increased cardiovascular risk in this population [9,10].

Recent trials suggest that nitrate-rich beetroot supplementation may enhance recovery-related cardiovascular adaptations, promoting more rapid post-exercise normalization of blood pressure and heart rate variability (HRV), particularly in older adults and populations experiencing vascular aging or autonomic impairment [4,5]. These effects are thought to be mediated by enhanced NO bioavailability and improved endothelial function. However, heterogeneity in study designs—including differences in nitrate dosage (1.5–20 mmol), duration (acute vs. chronic), exercise modality (aerobic, resistance, isometric), and HRV analysis methods—may influence observed outcomes. Additionally, reduced NO bioavailability has been linked to diminished physical function [11], raising the question of whether restoring NO levels through supplementation can enhance recovery and performance in postmenopausal women.

Given this complexity, systematic reviews and meta-analyses are essential for consolidating evidence across trials, estimating pooled effects, and identifying sources of variability. Utilizing frameworks, such as PRISMA and GRADE, such reviews can offer clinically meaningful conclusions and inform tailored interventions for populations in need—such as postmenopausal women, who may benefit from accessible, non-pharmacological strategies to enhance cardiovascular resilience [12,13].

Although previous reviews have explored dietary supplementation and performance, they have often focused narrowly on energy metabolism or endurance outcomes, without examining how supplementation affects physiological responses across different exercise types and population subgroups. Notably, earlier reviews [14,15,16,17,18] lacked an integrated approach comparing endurance and resistance contexts or addressing clinical heterogeneity in depth. To fill this gap, the present systematic review and meta-analysis evaluate the effects of beetroot supplementation on post-exercise cardiovascular and autonomic recovery in postmenopausal women.

While numerous reviews have explored the effects of dietary supplementation on athletic performance—often emphasizing isolated variables such as energy metabolism, endurance, or recovery—there remains a significant evidentiary gap concerning how such supplementation influences skeletal muscle contractile efficiency and oxygen utilization across different exercise modalities and clinical populations. Although prior reviews [14,15,16,17,18] have provided valuable insights into general performance outcomes, they typically lack a comprehensive comparative analysis between endurance and strength-based contexts, and rarely account for the heterogeneity observed across subpopulations.

Notably, quantitative syntheses focusing specifically on postmenopausal women are exceptionally scarce, despite the heightened cardiovascular risk and physiological complexity associated with this group. Most available literature either excludes this population or aggregates them with broader cohorts, obscuring sex- and age-specific responses to supplementation. This lack of focused meta-analytic evidence limits the development of targeted, evidence-based interventions for this vulnerable demographic.

In response, the present systematic review and meta-analysis aims to address this critical gap by quantitatively evaluating the effects of beetroot supplementation on post-exercise cardiovascular and autonomic recovery in postmenopausal women. By applying rigorous synthesis methods, this work seeks to generate clinically meaningful insights and clarify under what conditions beetroot-derived dietary nitrate may offer the greatest benefit to this underrepresented group.

## 2. Materials and Methods

This review was completed in accordance with the guidelines outlined in the Preferred Reporting Items for Systematic Reviews and Meta-Analyses (PRISMA) statement [12] and has been registered in the PROSPERO database under the identifier CRD42024619635.

### 2.1. Eligibility Criteria

The studies were obtained from peer-reviewed scientific journals, covering publications indexed from the inception of each database through July 2025. Selection followed predefined inclusion and exclusion criteria based on the PICOS framework (Population, Intervention, Comparison, Outcomes, and Study Design) (Table 1):

### 2.2. Information Source, Search Strategy, and Study Selection

The literature search was performed by means of EMBASE, LILACS, CINAHL, MEDLINE/PubMed (National Library of Medicine), Scopus, and Web of Science databases. The search strategy applied the following keywords: “Beetroot supplementation” OR “beetroot juice” OR “beetroot extract” OR “dietary nitrate” AND “Postmenopausal women” OR “post-menopause” OR “menopause” AND “Exercise” OR “Physical Activity” OR “Strengthening Program” OR “Training” OR “Rehabilitation” OR “Habilitation” OR “heart rate variability” (see Appendix A Table A1).

All identified articles were imported into the Rayyan QCRI platform (Qatar Computing Research Institute, Qatar) for duplicate elimination. Screening was attained within Rayyan by evaluating titles and abstracts.

For full-text evaluations, two reviewers (Reviewer 1 and Reviewer 2) independently assessed each article against the pre-established eligibility criteria. Reviewer 1 conducted the initial eligibility screening, while Reviewer 2 verified the decisions by re-evaluating the same full texts. Discrepancies were resolved through discussion. If consensus could not be reached, a third reviewer (Reviewer 3) was consulted to adjudicate and provide a final decision.

Once the final set of references was determined, the entire research team reviewed the selection and collectively judged the feasibility of conducting a meta-analysis.

### 2.3. Data Collection and Data Extraction

Information about the author, study design, participant characteristics, interventions, and exercise protocols of each included study was extracted from the primary sources and summarized in a table (Table 2). When obligatory, absent information was requested by contacting the corresponding authors. This process was performed independently by at least two reviewers. If no response was received from the study’s corresponding author, data presented in graphical format were retrieved via Web Plot Digitizer^®^—Version 4. Cardiovascular outcomes were stated as means and standard deviations (SD). For studies providing results as “standard error” or “confidence intervals” (CI), values were converted to SD.

### 2.4. Data Items

Data on cardiovascular parameters (e.g., systolic and diastolic blood pressure, vascular function) and physical performance indicators (e.g., VO_2max_, functional capacity) were recorded to enable comparisons between pre- and post-intervention outcomes. Information related to contributor characteristics, intervention details, and funding sources was extracted from the included studies. Any absent or unclear data were excluded from the analysis.

### 2.5. Assessment of the Risk of Bias in Individual Studies and Across Studies

The risk of bias assessment was conducted using the Cochrane Risk of Bias tool [12] within the Review Manager software (RevMan 5.4.1). This tool evaluates potential bias across six domains: randomization process, deviations from intended interventions, missing outcome data, measurement of the outcome, selection of the reported results, and overall bias. Each domain was classified as presenting low risk, some concerns, or high risk, based on the guidance from Sterne et al. (2019) [29], in addition to the reviewers’ informed judgment.

The evaluation was independently performed by two reviewers. Reviewer 1 was responsible for the initial domain-level assessment, assigning risk levels based on predefined criteria. Reviewer 2 independently reviewed each of those judgments for consistency and accuracy. In the event of disagreement between the two reviewers, a third reviewer acted as an arbitrator to resolve conflicts and finalize the classification. All reviewers underwent formal training on the Cochrane Risk of Bias tool before initiating the assessments to ensure consistency and methodological rigor.

Furthermore, potential overarching sources of bias affecting the evidence base—such as publication bias and selective outcome reporting—were evaluated via the same structured process

### 2.6. Certainty Assessment (Levels of Evidence)

The Grades of Recommendation, Assessment, Development, and Evaluation (GRADE) framework [13] was applied to assess the certainty of the evidence. This approach started with randomized trials being categorized as high-certainty evidence, with subsequent alterations based on factors such as study quality (clarity and rigor of methodology and execution) and limitations that could weaken the strength of the evidence [13]. The summary of findings table was created with GRADEpro GDT v4^®^ software Version 2022 (McMaster University, Hamilton, ON, Canada).

### 2.7. Qualitative Analysis (Systematic Review)

A narrative synthesis was completed to present detailed information on how each study was conducted. Study-specific findings were reported in both textual descriptions and tabular form. The qualitative analysis for each individual study focused on evaluating cardiovascular parameters within the intervention and control protocols.

### 2.8. Synthesis of Results and Summary Measures

Following the final selection of eligible studies, the feasibility of conducting a quantitative synthesis through meta-analysis was assessed. When meta-analysis was deemed appropriate, outcome values were extracted based on post-intervention data, prioritizing between-group comparisons. If necessary data were missing—such as standard deviations (SD), 95% confidence intervals (CI), standard errors (SE), or *p*-values—SDs were estimated from other dispersion measures following Cochrane guidelines.

Effect sizes were calculated using weighted mean differences (MDs) with corresponding 95% confidence intervals (CIs). A random-effects model was applied in all analyses to account for both within-study and between-study variability, recognizing the expected methodological and clinical heterogeneity among included trials. Statistical significance was established at *p* < 0.05 for the overall MD comparing the intervention and control groups.

Heterogeneity was assessed via the I^2^ statistic, with interpretation as follows [30,31,32]:

0–29%: might not be important.

30–49%: may represent moderate heterogeneity.

50–74%: may represent substantial heterogeneity.

75–100%: may indicate considerable heterogeneity.

Where substantial heterogeneity (I^2^ ≥ 50%) was detected, subgroup analyses were planned a priori to explore differences by:

Type of exercise intervention (e.g., aerobic vs. resistance).

Mode of beetroot supplementation (e.g., juice vs. capsule).

Population characteristics (e.g., age range, presence of comorbidities).

Additionally, if necessary, it was prearranged to conduct sensitivity analyses by sequentially excluding each study to determine its impact on overall estimates and heterogeneity. Where data sufficed, meta-regression was anticipated to explore the influence of moderator variables (e.g., intervention duration, dosage of nitrate, baseline fitness level) on effect sizes. Yet, if only one reference remained, it was not permissible to reconduct meta-analysis.

To assess publication bias, funnel plots were visually inspected when ≥10 studies were included in a single outcome analysis. In cases with asymmetry, Egger’s test was planned to statistically evaluate small-study effects.

For heart rate variability (HRV) outcomes specifically, the first post-exercise recovery measurement was selected, as it is considered to offer greater external validity and ecological relevance in real-world physical activity settings.

All statistical analyses were conducted using Review Manager (RevMan) version 5.4.1, and methods adhered to PRISMA and Cochrane Handbook guidelines.

## 3. Results

### 3.1. Study Selection

**Figure 1:**
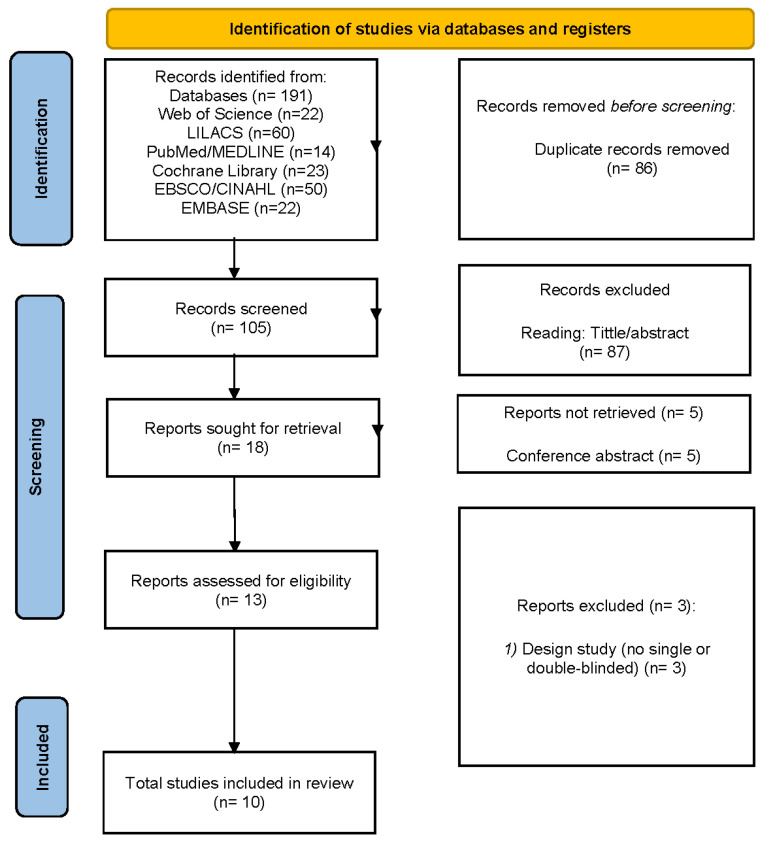
PRISMA 2020 flow diagram for new systematic reviews, which included searches of databases and registers only.

Figure 1 illustrates the process of identifying, screening, and selecting studies for inclusion in a systematic review. The process started with the identification of 191 records from database searches. Of these, 86 records were omitted before screening owing to duplication, leaving 105 records for screening. During the screening phase, 87 records were excluded based on title and abstract assessment, resulting in 13 reports identified for retrieval. In the eligibility assessment, three reports were excluded for not conducting a placebo design. Hence, a total of 10 studies satisfied the eligibility criteria and were included in the systematic review.

### 3.2. Study Characteristics

In a double-blind, randomized crossover placebo-controlled trial, Amaral et al. (2019) [19] scrutinized whether acute beetroot juice (BJ) ingestion could enhance post-exercise hypotension (PEH) in hypertensive postmenopausal women. The hypothesis was that BJ would increase nitric oxide (NO) bioavailability, assessed via salivary nitrite, and amplify blood pressure reductions after aerobic exercise. Thirteen physically active postmenopausal women with hypertension (58.1y (4.6); BMI 27.4 kg/m^2^ (4.2)), all prescribed antihypertensive medication (excluding beta-blockers), completed the trial. Eligibility included confirmed hypertension, menopause, and exercise capacity. Exclusion criteria were hormone therapy, nitrate sensitivity, diabetes, cardiovascular events, and smoking. Participants completed 40 min of moderate treadmill exercise (65–70% HRR) in three separate sessions, each preceded by a different drink consumed two hours before exercise: BJ (400 mg nitrate), a nitrate-depleted placebo (PLA), and an orange-flavored control (OFD). Sessions were separated by a minimum five-day washout.

The identical procedure was enforced in another publication, Amaral et al. (2021) [20], where the researchers investigated antioxidant responses after aerobic exercise. The beverages were consumed two hours before exercise, and saliva samples were logged at four time points: at baseline (0′), 120 min after beverage ingestion (130′), immediately after exercise (170′), and 90 min post-exercise (260′). The primary outcome trials included salivary total protein, catalase activity, reduced glutathione (GSH), and total antioxidant capacity evaluated by the Ferric Reducing Antioxidant Power (FRAP) assay.

Caldwell et al. (2019) [23] led a randomized, double-blind, placebo-controlled crossover trial to assess the acute effects of dietary nitrate on vascular control through exercise in hypertensive postmenopausal women. The study aimed to determine whether a single dose of nitrate could lower resting and exercise blood pressure, enhance forearm blood flow (FBF), and improve functional sympatholysis. Ten sedentary postmenopausal women presented with hypertension (56 ± 1 years; BMI 31 ± 3 kg/m^2^) contributed. Most physical activity involved walking. Medication regimens included combinations of angiotensin-II blockers, diuretics, beta-blockers, and calcium channel blockers. Participants maintained their usual pharmacotherapies and were told to avoid alcohol, mouthwash, and chewing gum 24 h before each session. The intervention included dynamic handgrip exercise at 20% of maximal voluntary contraction (MVC) for 7 min. A cold pressor test (CPT) was applied during minutes 5 to 7 of the protocol. Blood pressure was verified continuously by finger photoplethysmography, and FBF was assessed using brachial artery ultrasonography. Two test beverages were ingested two hours before exercise: a nitrate-rich beetroot juice (12.9 mmol nitrate from 140 mL Beet It Sport) and a nitrate-depleted placebo version. Session phases were randomized.

Benjamim et al. (2024) [21] conducted a triple-blinded, randomized, placebo-controlled, crossover trial to evaluate the acute and short-term effects of nitrate-rich beetroot juice on cardiovascular and autonomic responses following aerobic exercise in hypertensive postmenopausal women. The study aimed to assess whether beetroot juice could enhance post-exercise reductions in blood pressure, improve endothelial function, and increase HRV. The trial enrolled 14 physically inactive postmenopausal women aged 59 ± 4 years, with a BMI of 29.2 ± 3.1 kg/m^2^ and medically diagnosed with hypertension. Physical inactivity was verified via the Modified Baecke Questionnaire (score < 9.11). Eligibility required women aged 50–65, cleared for exercise by the Physical Activity Readiness Questionnaire (PAR-Q). Participants with cardiovascular events, allergies to nitrate, milk or gluten, or prescribed medications such as beta-blockers and hormone therapy were omitted. Participants completed 30 min of submaximal aerobic exercise on a treadmill (65–70% VO_2peak_) under two protocols: an acute intervention involving a single 800 mg dose of nitrate (140 mL beetroot juice), and a short-term protocol with 400 mg/day for six days (70 mL/day). The placebo juice, identical in appearance and taste, was nitrate-depleted (−94%) by an ion exchange resin. Both interventions were administered in randomized order with 7 days of washout between phases.

Caldwell et al. (2024) [24] performed a randomized, double-blind, placebo-controlled, crossover trial to explore the acute effects of inorganic nitrate supplementation on vascular and blood pressure responses during incremental handgrip exercise in postmenopausal women. The study hypothesized that nitrate would reduce blood pressure but not significantly alter endothelial function. Twelve healthy postmenopausal women (64 ± 5 years; BMI 26 ± 6 kg/m) participated. All were free of cardiovascular diseases, hypertension, hormone therapy, and use of proton pump inhibitors. Participants were instructed to maintain their usual diet, avoid mouthwash, and fast for four hours before each session. The exercise protocol included handgrip effort at 10%, 15%, and 20% of maximal voluntary contraction (MVC), each sustained for 3 min. Macro- and microvascular function were judged using flow-mediated dilation (FMD), ischemic FMD, and post-occlusive reactive hyperemia (PORH) via near-infrared spectroscopy (NIRS). Testing sessions were separated by 48 h washouts. Participants consumed either nitrate-rich beetroot juice (140 mL, ~12.9 mmol nitrate) or a nitrate-poor blackcurrant placebo (<0.2 mmol nitrate) two hours before testing, administered in randomized order.

Carrijo et al. (2021) [25] directed a randomized, double-blind, placebo-controlled, crossover trial to evaluate whether a single dose of beetroot juice could enhance HRV following moderate-intensity aerobic exercise in hypertensive postmenopausal women. The study compared high-nitrate, low-nitrate, and placebo drinks consumed before exercise. Thirteen women aged 58.1 ± 4.6 years (BMI 27 ± 4 kg/m^2^) with controlled hypertension and in post menopause were included. Eligibility required a non-smoking status, no hormone therapy or beta-blockers, and the ability to perform treadmill exercise. Exclusion criteria included a history of cardiovascular events, diabetes, food allergies, and nitrate sensitivity. The exercise intervention consisted of 40 min of continuous treadmill walking at 65–70% of HRR, including warm-up and cool-down. Testing sessions were divided by at least seven days. Participants consumed one of three beverages two hours before each session. The drinks included: (1) High-nitrate beetroot juice (400 mg nitrate from 35 mL Beet-It Sport diluted in 315 mL water), (2) low-nitrate beetroot juice filtered to 3.86 mmol/kg nitrate, and (3) a non-caloric orange-flavored placebo. All drinks were matched in volume and flavor and administered in a randomized order.

Hogwood et al. (2023) [26] performed a randomized, double-blind, placebo-controlled to investigate the acute effects of exercise intensity and nitrate-rich beetroot juice (BRJ) supplementation on vascular function in postmenopausal women. The study equated flow-mediated dilation (FMD) and vascular biomarkers following moderate- and high-intensity exercise with and without nitrate. Twenty-four postmenopausal women (ages 59–61, BMI 27–28 kg/m^2^) were randomly assigned to BRJ (*n* = 12) or placebo (*n* = 12) groups. Inclusion required participants to be at least one year postmenopausal, sedentary or recreationally active, non-smokers, and free from hormone therapy. Exclusion criteria included the use of medications affecting nitrate metabolism and prior hysterectomy or ovariectomy. The protocol involved three test sessions: (1) High-intensity exercise (HIE) at 75% Δ between LT and VO_2peak_, (2) Moderate-intensity exercise (MIE) at lactate threshold power output, and (3) Passive rest (Control). Each session was standardized to 200 kcal of energy expenditure. Supplements were consumed for two days before testing and again two hours prior to each session.

Participants ingested 140 mL of either BRJ (~13 mmol nitrate) or nitrate-depleted placebo (<0.1 mmol nitrate) in a blinded manner. Blood samples were taken pre- and post-exercise to assess plasma nitrate, nitrite, and endothelin-1 (ET-1) levels.

Proctor et al. (2022) [27] performed a double-blind, randomized, placebo-controlled, crossover trial to assess the acute effects of dietary nitrate on exercise tolerance during handgrip activity under blood flow restriction in postmenopausal women. The study hypothesized that nitrate supplementation would enhance performance under ischemic conditions by improving time-to-fatigue and force output. Thirteen postmenopausal women aged 57–76 years with reduced nitric oxide bioavailability were included. Participants were excluded if they engaged in regular exercise (>3 times/week), had major chronic diseases, abnormal BMI (<18.5 or >35 kg/m^2^), anemia, organ dysfunction, or had participated in a recent blood donation. Use of tobacco, cardiovascular drugs, or hormone therapies was likewise exclusionary. Participants completed rhythmic isometric handgrip contractions at 10% MVC (30 reps/min), with progressive forearm ischemia induced by cuff inflation (increased by 20 mmHg per minute) until volitional fatigue. The protocol was repeated during two visits separated by at least seven days. Each contributor consumed either nitrate-rich beetroot juice (~9.7 mmol nitrate in 140 mL Beet-It Organic) or a nitrate-depleted placebo 100 min prior to testing. Plasma nitrate and nitrite concentrations were measured pre- and post-ingestion to confirm systemic nitrate uptake.

In a randomized, double-blind, placebo-controlled, crossover clinical trial, Benjamim et al. [22] judged the effects of dietary nitrate ingestion on physical performance tests in postmenopausal women aged 50–65 years. The study proposed to explore whether an eight-day protocol of beetroot juice supplementation could improve physical performance outcomes, mostly aerobic capacity and muscle strength, in postmenopausal women. The trial included 15 postmenopausal women with low physical activity levels as evaluated by the Baecke Modified Questionnaire for older adults (mean score: 4.43 ± 1.84). The mean age of participants was 59 ± 4 years, with a BMI of 29.3 ± 3 kg/m^2^. Inclusion criteria required contributors to be postmenopausal women between 50 and 65 years of age, not undertaking hormone replacement therapy, and free of cardiovascular diseases such as myocardial infarction, stroke, or heart failure. Exclusion criteria included the use of proton pump inhibitors, smoking, and recent engagement in intense physical activity. The intervention consisted of two branches: (1) Nitrate-rich Beetroot Juice (BRJ-NO^3−^) containing approximately 400 mg of nitrate from 70 mL of beetroot juice; and (2) Placebo (PLA), which was nitrate-depleted beetroot juice with an equivalent appearance and taste. Participants ingested one bottle per day for eight consecutive days for each intervention, with a minimum washout period of seven days between protocols. They were instructed to consume the beetroot juice in the morning prior to brushing their teeth to avoid interference with nitrate metabolism.

The primary purpose of the Zoughaib et al. (2023) [28] study was to determine whether advances in muscle speed and power observed after acute ingestion of nitrate-rich BRJ in older individuals would be upheld after short-term supplementation, daily for 2 weeks. Secondarily, the study sought to confirm whether short-term BRJ supplementation would be linked with changes in blood pressure or plasma markers of oxidative stress in this population. Participants were assigned to one of two groups: BRJ with nitrate (NO^3−^) or BRJ without nitrate (placebo). Each contributor was considered after acute ingestion and again after 2 weeks of daily supplementation, followed by a 2-week washout period, before being crossed over to the other treatment. Participants consumed 140 mL of concentrated BRJ, containing 18.2 ± 6.2 mmol of NO^3−^ (nitrate group) or <0.05 mmol of NO^3−^ (placebo group). Sixteen older individuals, residing in the community, aged 65 to 79 years (mean 71 ± 5 years), 5 men and 11 women, participated in the study. Pregnant or lactating women, smokers, those with anemia, those consuming certain medications (proton pump inhibitors, antacids, xanthine oxidoreductase inhibitors, phosphodiesterase inhibitors, hormone replacement therapy), and those with a history of metabolic, cardiovascular, renal, neuromuscular, or hepatic diseases were excluded. They were instructed to avoid nitrate-rich foods (spinach, arugula, beets) for 24 h, in addition to alcohol, caffeine, or food for 12 h before each study visit. They were instructed not to use antibacterial mouthwash. Blood pressure was logged, and blood samples were collected periodically during each ~3 h experiment. Muscle function was assessed using isokinetic dynamometry. An important issue that needs to be considered is that this study also included men.

### 3.3. Results of Individual Studies

Amaral et al. (2019) [19] documented that acute beetroot juice ingestion significantly increased salivary nitrite (NO^2−^) levels when equated to a placebo and a non-caloric orange-flavored drink. Salivary NO^2−^ concentration was highest instantly after exercise (3.3 mM) and remained elevated 90 min post-exercise (2.5 mM) when compared to placebo (0.9 mM) and the orange-flavored drink (0.1 mM) (*p* < 0.01). Nonetheless, despite the bigger salivary NO^2−^, systolic and diastolic blood pressure (SBP and DBP) decreased post-exercise across all sessions, but no changes were observed between the interventions (*p* = 1.000).

Amaral et al. (2021) [20] documented that beetroot juice ingestion significantly reduced the salivary catalase activity after exercise in both high-nitrate and low-nitrate conditions compared to placebo (*p* < 0.001). Similarly, the area under the curve (AUC) of reduced glutathione (GSH) was significantly lower after high-nitrate beetroot juice ingestion compared to the placebo (*p* < 0.001). No significant changes were noticed in total antioxidant capacity evaluated by the Ferric Reducing Ability of Plasma (FRAP) among the experimental sessions.

Benjamim et al. [21] found significant reductions in systolic blood pressure (SBP) following acute ingestion of beetroot juice nitrate-rich (BRJ-NO^3−^ rich), with a decrease of −9.28 mmHg (95% CI: −1.68 to −16.88) compared to placebo. Flow-mediated dilation (FMD) values increased acutely after exercise (3.18% (0.36 to 5.99), *p* = 0.031) and after a one-week intervention (4.2% (1.52 to 6.87), *p* = 0.004) compared to placebo. HRV indices demonstrated quicker recovery of parasympathetic modulation in the BRJ-NO^3−^ rich group. Yet, no differences were identified in heart rate recovery between groups. One-week BRJ-NO^3−^ rich ingestion did not decrease blood pressure post-exercise, although it improved FMD and the recovery of parasympathetic cardiac modulation biomarkers.

Benjamim et al. [22] documented that eight days of beetroot juice nitrate-rich (BRJ-NO^3−^ rich) ingestion significantly increased nitrite (NO^2−^) plasma concentrations (0.41 μM vs. 0.18 μM, *p* < 0.001) and nitrate (NO^3−^) plasma concentrations (382 μM vs. 74 μM, *p* < 0.001) compared to placebo. The 6 min walk test (6 MWT) distance was significantly improved in the BRJ-NO^3−^ rich group by 19.6 m (95% CI: 1.33 to 37.88), *p* = 0.038. Still, other physical performance tests, such as arm curl, sit-to-stand, and agility tests, did not reveal significant differences between conditions.

Caldwell et al. (2019) [23] recognized that acute nitrate-rich (NR) supplementation significantly enlarged plasma nitrite levels (809 nM vs. 79 nM, *p* < 0.001) compared to placebo. Functional sympatholysis improved by about 50% during steady-state exercise after NR supplementation. Hitherto, resting systolic blood pressure (SBP), diastolic blood pressure (DBP), and mean arterial pressure (MAP) were not significantly different between conditions. Likewise, forearm blood flow (FBF) during steady-state exercise was significantly reduced in the NR condition, equated to placebo (190 mL/min vs. 218 mL/min, *p* = 0.03).

Caldwell et al. (2024) [24] recognized that acute nitrate-rich (NR) supplementation significantly diminished aortic systolic blood pressure (112 ± 11 mmHg vs. 118 ± 10 mmHg, *p* = 0.005), brachial systolic blood pressure (120 ± 12 mmHg vs. 127 ± 12 mmHg, *p* = 0.002), and mean arterial pressure (87 ± 10 mmHg vs. 90 ± 9 mmHg, *p* = 0.022) linked to nitrate-poor (NP) supplementation. Yet, no significant differences were apparent in flow-mediated dilation (FMD) or post-occlusive reactive hyperemia (PORH) measures between conditions. Moreover, forearm blood flow and vascular conductance during incremental handgrip exercise were not significantly different between the NR and NP conditions.

Carrijo et al. (2021) [25] recognized that a single dose of beetroot juice, regardless of nitrate (NO^3−^) content, did not modify aerobic exercise-mediated responses in HRV in hypertensive postmenopausal women. No significant changes were revealed in HRV indexes across time, frequency, or non-linear domains when equating high-NO^3−^, low-NO^3−^, and placebo conditions. Yet, moderate aerobic exercise itself promoted increases in HRV indexes, particularly in frequency and non-linear domains, suggesting exercise alone improves HRV in this cohort without the additional benefits from beetroot juice ingestion.

Hogwood et al. (2023) [26] documented that inorganic nitrate supplementation (BRJ) significantly increased plasma nitrate and nitrite levels and decreased endothelin-1 (ET-1) compared to placebo. BRJ promoted peak flow-mediated dilation (FMD) post-exercise associated with placebo (*p* < 0.05), mostly after high-intensity exercise (HIE). The combination of BRJ and HIE improved FMD over time (*p* = 0.05), while BRJ with moderate-intensity exercise (MIE) resulted in medium effects. No significant improvements were recognized for blood pressure or pulse wave velocity across all conditions.

Proctor et al. (2022) [27] documented that acute nitrate-rich (BR) supplementation significantly improved plasma nitrate (16.2-fold) and nitrite (4.2-fold). Time to volitional fatigue through ischemic handgrip exercise was protracted by 61.8 ± 56.5 s (*p* = 0.003) after BR ingestion. Also, nitrate supplementation improved the rate of force development throughout progressive muscle ischemia (*p* = 0.023) between 50% and 75% of the time to fatigue. Yet, no significant effects were detected for the mean duration of contraction, relaxation rates, or rating of perceived effort (RPE) between conditions.

The Zoughaib et al. (2023) [28] study concludes that nitrate-rich BRJ supplementation, both acutely and in the short term, provides similar benefits on muscle speed and power in elderly men and women, but does not affect blood pressure or plasma oxidative stress.

### 3.4. Risk of Bias

**Figure 2:**
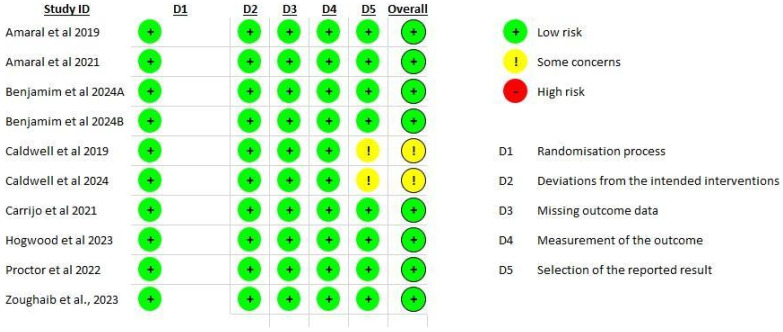
Bias risk analysis (RoB 2) [19,20,21,22,23,24,25,26,27,28].

The assessment of bias risk differed amongst the studies included, with certain concerns recognized in areas like the reporting of results. Typically, most studies revealed a low risk of bias, with only some presenting minor concerns (Figure 2). Detailed information is provided in Appendix A Table A1.

### 3.5. Randomization Process

All ten studies (100%)—Amaral et al. (2019) [19], Amaral et al. (2021) [20], Benjamim et al. (2024) [21], Benjamim et al. (2024) [22], Caldwell et al. (2019) [23], Caldwell et al. (2024) [24], Carrijo et al. (2021) [25], Hogwood et al. (2023) [26], Proctor et al. (2022) [27], and Zoughaib et al. (2023) [28], clearly defined the randomization process, including appropriate sequence generation and allocation concealment. Thus, all studies were rated as low risk for this domain.

### 3.6. Deviations from Intended Interventions

All studies (100%) were judged as low risk of deviations from the intended interventions. They satisfactorily stated the blinding of participants and investigators or implemented protocols ensuring that changes were not influenced by the trial setting. Also, suitable statistical analyses were completed to account for any potential differences.

### 3.7. Missing Outcome Data

All studies (100%) established low risk concerning absent outcome data. They either reported complete outcome data or satisfactorily addressed missing data through appropriate statistical approaches, ensuring that any missing information did not influence the stated results.

### 3.8. Measurement of Outcomes

All studies (100%) were rated as low risk regarding outcome measurement, as they reliably enforced suitable and standardized methods for evaluating outcomes relevant to the interventions being tested. Equally, outcome assessors were either adequately blinded or the nature of the outcomes measured minimized the probability of detection bias. The consistency in measurement techniques across the studies further maintained the reliability of the findings.

### 3.9. Selection of Reported Results

Most studies (78%) were judged as low risk for the selection of reported results. Yet, the studies of Caldwell et al. (2019) [23] and Caldwell et al. (2024) [24] raised concerns in this domain, due to a lack of clear pre-specified analysis plans, increasing the prospect of selective reporting.

### 3.10. Overall Bias

The overall risk of bias was low for most studies (78%), including Amaral et al. (2019, 2021) [19,20], Benjamim et al. (2024) [21,22], Carrijo et al. (2021) [25], Hogwood et al. (2023) [26], Zoughaib et al. (2023) [28], and Proctor et al. (2022) [27]. Still, Caldwell et al. (2019) [23] and Caldwell et al. (2024) [24] offered some concerns generally because of issues related to the selection of reported results. This qualitative analysis indicates that the majority of the studies offer a robust methodological approach with low risk of bias, while some require careful interpretation owing to potential methodological concerns.

### 3.11. Synthesis of Results

**Figure 3:**
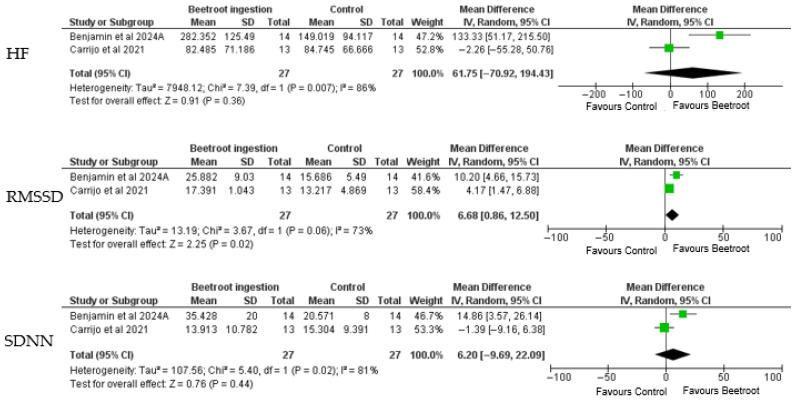
Meta-analysis for the overall effects of beetroot on HF, RMSSD, and SDNN following exercise. HF: High frequency; RMSSD: Root Mean Square of Successive Differences; SDNN: Standard Deviation of Normal-to-Normal Intervals. (RMSSD) Root Mean Square of Successive Differences; (HF) High Frequency (power of heart rate variability); SDNN Standard Deviation of Normal-to-Normal Intervals. Green square: Primary studies. Black diamonds: Amounted studies [21,25].

Figure 3 illustrates forest plots evaluating the effects of beetroot ingestion on heart rate variability (HRV) parameters, including high-frequency (HF) power, root mean square of successive differences (RMSSD), and the standard deviation of normal-to-normal intervals (SDNN), based on data from two randomized controlled trials [21,25].

For HF power, the pooled analysis included 27 participants in each group. The mean difference was 61.75 ms^2^ (95% CI: −70.92 to 194.43) in favor of beetroot ingestion; however, the result did not reach statistical significance (*p* = 0.36). The analysis revealed considerable heterogeneity (I^2^ = 86%, *p* = 0.007), suggesting marked variability in responses between studies. Clinically, while HF reflects parasympathetic (vagal) activity, the wide confidence interval and inconsistency weaken the strength of the inference.

In contrast, RMSSD, a reliable time-domain marker of vagal modulation, showed a statistically significant improvement with beetroot supplementation. The pooled mean difference was 6.68 ms (95% CI: 0.86 to 12.50, *p* = 0.02), favoring the intervention. Although substantial heterogeneity was present (I^2^ = 73%, *p* = 0.06), both studies demonstrated a consistent direction of effect. Clinically, an increase of ~6.7 ms in RMSSD suggests enhanced post-exercise parasympathetic reactivation, which may indicate more efficient cardiovascular recovery—particularly relevant in populations with autonomic impairment, such as postmenopausal women.

Regarding SDNN, the overall mean difference was 6.20 ms (95% CI: −9.69 to 22.09), which was not statistically significant (*p* = 0.44). Heterogeneity was again high (I^2^ = 81%, *p* = 0.02). Benjamim et al. [22] observed a notable increase in SDNN following beetroot ingestion, while Carrijo et al. [25] did not replicate this effect. Clinically, although SDNN reflects overall HRV and combined autonomic input, the lack of statistical significance and inconsistency across studies limit its interpretive value in this context.

In summary, the most robust and clinically relevant finding was the significant increase in RMSSD, supporting the hypothesis that beetroot-derived nitrates may facilitate autonomic recovery post-exercise via enhanced vagal tone. However, variability in results across HRV domains highlights the need for further trials with standardized protocols and larger samples.

### 3.12. GRADE Assessment

Table 3 presents the certainty of evidence around the effects of beetroot supplementation compared to placebo on three HRV indices: HF, RMSSD, and SDNN. The assessment is based on two randomized controlled trials, including a total of 54 participants (27 per group).

The certainty of evidence for HF is very low. While the studies were randomized trials with no serious risk of bias or indirectness, the analysis exhibited severe inconsistency (I^2^ = 86%) and very serious imprecision, as reflected by a wide 95% confidence interval (MD 61.75 ms^2^, 95% CI: −70.92 to 194.43). The magnitude and direction of the effect remain indeterminate. All plausible residual confounding would diminish the observed effect.

The evidence for RMSSD was graded as moderate. The certainty of evidence for SDNN is low. Although the studies were randomized trials with no serious risk of bias or indirectness, the analysis demonstrated very serious inconsistency, indicating substantial heterogeneity among the results. Also, there was serious imprecision, probably because of small sample sizes or wide confidence intervals that prevent firm conclusions. These limitations contribute to uncertainty regarding the true magnitude and direction of the effect.

The results suggest a possible beneficial impact of beetroot ingestion on HRV—particularly RMSSD—but highlighted the need for further well-constructed trials with standardized protocols to reduce heterogeneity and advance precision.

## 4. Discussion

It was proposed to measure the effects of beetroot on post-exercise recovery in postmenopausal women through a systematic review and meta-analysis. As main data, it was described that:

The meta-analysis established that beetroot impacts RMSSD HRV index, but serious concerns were recognized for risk of bias, indirectness, or imprecision; the evidence exhibited very serious inconsistency (I^2^ = 73%). Despite this, the pooled estimate (MD 6.68 ms, 95% CI: 0.86 to 12.50) advocates a beneficial effect of beetroot ingestion. The presence of heterogeneity does not negate the conceivable clinical relevance of this outcome.

The certainty of evidence for SDNN was rated as low. Although the trials were well-designed with no significant concerns for risk of bias or indirectness, the results offered serious inconsistency (I^2^ = 81%) and serious imprecision (MD 6.20 ms, 95% CI: −9.69 to 22.09), both of which limit confidence in the estimation. As with other outcomes, plausible residual confounding is expected to diminish the observed effect. RMSSD had a higher value in the beetroot group, suggesting improvements directed by beetroot compounds on parasympathetic reactivation.

(1) Risk of bias specified some concerns for the selection of the reported result, but demonstrated low risk for the residual items.
(2) GRADE established very low inevitability for HF, low certainty for SDNN, and moderate certainty for RMSSD.

Dietary nitrate, principally found in vegetables such as beetroot, performs a central role in modulating cardiovascular function during and after exercise by its conversion to nitric oxide (NO), a bioactive signaling molecule. Upon digestion, nitrate (NO^3−^) is absorbed in the small intestine, secreted in saliva, and then reduced to nitrite (NO^2−^) by oral bacteria before being further converted to NO through enzymatic and non-enzymatic mechanisms in hypoxic tissues [1]—conditions prevalent during exercise and early recovery phases [33,34]. Nitric oxide exerts several physiological effects conducive to cardiovascular recovery, including vasodilation, improved endothelial function, and reduction in systemic vascular resistance, all of which can permit more speedy blood pressure normalization post-exercise [21,22]. In addition, NO contributes to autonomic regulation by enhancing parasympathetic reactivation, which is a critical mechanism underlying post-exercise HRV recovery [34]. Chronic or acute nitrate supplementation has also been related to a reduction in post-exercise inflammatory markers and oxidative stress through its influence on oxylipin pathways and decrease in complement activation proteins, further supporting its role in promoting cardiovascular homeostasis after physical exertion [33]. Together, these mechanisms suggest that nitrate supplementation can increase cardiovascular resilience, explicitly in populations with impaired endothelial or autonomic function.

The statistically significant increase in RMSSD after beetroot supplementation indicates a possible advantage for autonomic recovery post-exercise. RMSSD reflects short-term variations in heart rate driven by vagal activity and has been related to enhanced cardiovascular resilience and recovery [35]. In the context of postmenopausal women—who characteristically experience diminished autonomic modulation [27]—this result supports the hypothesis that nitrate-rich beetroot may partly restore parasympathetic tone, maybe through enhanced nitric oxide bioavailability.

Despite promising trends, the meta-analytic estimates for HF and SDNN did not reach statistical significance. These parameters are similarly affected by vagal input (HF) and overall autonomic modulation (SDNN) [35]; hitherto, they may be more sensitive to inter-individual variability or confusing factors not uniformly controlled across trials. Additionally, SDNN may require longer recording periods or chronic interventions to capture significant changes, which most of the included studies did not consider.

Similarly, the relatively short duration of HRV recordings and the acute nature of most interventions may have restricted the ability to detect sustained autonomic adaptations. Assuming that HF and SDNN reflect both transient and cumulative autonomic influences, their sensitivity may depend on more prolonged exposure to dietary nitrate or repeated exercise bouts over time [35,36]. Future clinical trials incorporating extended monitoring periods and longer supplementation protocols are hence vital to fully explain the potential benefits of beetroot on these complex HRV domains.

### 4.1. Dose Considerations

The results of this review propose that the dose of dietary nitrate delivered through beetroot supplementation could be a determinant of its physiological efficacy in postmenopausal women. Across the included studies, nitrate doses ranged from about 1.5 mmol to 20 mmol, with most trials utilizing standardized beetroot juice shots containing either 400 mg or 800 mg of nitrate. Although higher doses were largely associated with greater elevations in plasma nitrate/nitrite levels and useful deviations in endothelial and autonomic parameters, results were not uniformly positive, mainly in studies with acute protocols or lower physical activity levels. These dose-related inconsistencies highlight the necessity for systematic dose–response trials, which are absent in this current population.

From a clinical perspective, understanding the optimal nitrate dose is vital for ensuring both efficacy and safety, particularly in women with comorbidities or prescribed antihypertensive medications. While no serious adverse events were specified, the possibility for hypotension or interactions with pharmacotherapies such as diuretics or angiotensin receptor blockers warrants caution. Also, for practical application, doses of about 400–800 mg/day appear reasonable and well-tolerated, mostly if administered in the form of commercially available concentrated beetroot juice.

In the context of postmenopausal women engaging in exercise, nitrate supplementation may help as a non-pharmacological adjunct to improve post-exercise vascular and autonomic recovery. Nonetheless, the present evidence does not hitherto allow for absolute clinical recommendations, predominantly given the variability in responses observed with acute versus short-term dosing. Consequently, future research should aim to establish evidence-based dosing protocols tailored to exercise modality, intensity, and individual health status. Thus, to better guide personalized and bespoke interventions for cardiovascular support in this susceptible population.

### 4.2. Limitations

Several limitations of this systematic review and meta-analysis should be documented. Firstly, the number of eligible trials was small (*n* = 10), and individual studies generally included modest sample sizes, which may restrict the statistical power and precision of pooled estimates. Secondly, most studies accepted short-term or acute supplementation protocols, thus restricting the extrapolation of results to long-term interventions or chronic health outcomes. Thirdly, generalizability is inhibited owing to the underrepresentation of women with multiple comorbidities and the exclusion of participants using medications—typically beta-blockers—that can significantly impact autonomic function and HRV metrics. Fourthly, while most trials were described as double-blind, the efficacy of blinding may have been compromised by the distinct taste and color of beetroot juice, and the potential for placebo effects was inadequately explored or controlled. Furthermore, heterogeneity in intervention protocols—e.g., nitrate dose, juice formulation, and exercise modality—combined with inconsistencies in HRV measurement methods and timing, could have contributed to the sizable between-study variability observed. These factors collectively emphasize the importance of methodological rigor and standardization in forthcoming scientific research on dietary nitrate interventions in postmenopausal populations.

An important constraint of this review was the extensive to substantial heterogeneity observed across pooled analyses (I^2^ from 73% to 86%). This could reflect changes in methodological and biological factors across the included studies. Initially, there were notable contradictions in the nitrate doses and formulations, ranging from 1.5 to 20 mmol of nitrate, and directed as concentrated beetroot shots, diluted juices, or nitrate-depleted placebos. Such deviations may influence nitrate absorption and nitric oxide bioavailability. Second, the duration of supplementation varied from single acute doses to short-term protocols lasting up to eight days, which may differentially affect endothelial and autonomic responses. Third, exercise protocols involved moderate-intensity aerobic training, rhythmic handgrip exercises, and high-intensity exertion, each of which elicits distinct cardiovascular and autonomic challenges. Fourth, participant features were heterogeneous in terms of physical activity levels, antihypertensive medication use (e.g., angiotensin receptor blockers, beta-blockers, diuretics), and the presence or absence of comorbidities—all can modify cardiovascular responses and confound HRV measurements. Finally, conflicts in HRV measurement methods and timing—including differences in recording duration, timing relative to exercise cessation, and analytical domains (time, frequency, and non-linear techniques) could have offered further variability and limited cross-study comparability. Together, these methodological differences highlight the imperative necessity for standardized protocols in impending trials assessing nitrate supplementation and post-exercise autonomic recovery in postmenopausal women.

### 4.3. Practical Implications

Despite the methodological limitations identified in this review, beetroot supplementation emerges as a promising, safe, and low-cost nutritional strategy to increase post-exercise autonomic recovery in postmenopausal women. Given its abundant nitrate content and potential to improve nitric oxide bioavailability, beetroot may help counteract age-and menopause-related declines in vascular function and parasympathetic modulation. This is particularly applicable for populations with sharp cardiovascular risk profiles, physical inactivity, or existing autonomic imbalance. Similarly, its natural origin and minimal adverse effects position beetroot as an appealing adjunct to exercise-based rehabilitation and preventive health strategies. However, the clinical application of beetroot supplementation should be acknowledged with caution, as small sample sizes, heterogeneous protocols, and short-term interventions limit the current body of evidence. Until larger, well-designed trials approve its efficacy and define optimal dosage, duration, and target populations, healthcare professionals should consider its use as complementary rather than substitutive to conventional interventions.

### 4.4. Future Research Directions

To further the evidence base about beetroot supplementation and cardiovascular recovery in postmenopausal women, forthcoming studies should prioritize more rigorous and standardized methodologies. First, there is a need to include larger and more diverse populations, including those with varied ethnic backgrounds, comorbidity profiles, and physical activity levels, to enhance generalizability. Second, longer-term supplementation protocols should be employed to evaluate the sustainability of cardiovascular and autonomic benefits over time. Third, trials must study dose–response relationships, determining the optimal nitrate concentration and frequency required to provoke meaningful physiological adaptations. Also, comparative studies of diverse exercise modalities and intensities (e.g., aerobics, resistance, high-intensity interval training) are needed to understand interaction effects with nitrate. Evaluating further physiological outcomes, such as inflammatory markers (e.g., IL-6, C-reactive protein), endothelial function (e.g., flow-mediated dilation), and oxidative stress biomarkers, will help explicate the broader mechanisms of action. Finally, the standardization of HRV assessment protocols—including the timing, duration, and analytical domains—and the incorporation of 24 h HRV monitoring could produce more comprehensive insights into autonomic regulation and recovery dynamics.

## 5. Conclusions

Beetroot supplementation, principally when combined with exercise, shows potential in enhancing autonomic recovery in postmenopausal women, as indicated by improvements in RMSSD. However, the evidence remains incomplete due to methodological heterogeneity and imprecision. Further high-quality randomized controlled trials are necessary to confirm these findings and clarify the clinical utility of beetroot in this population.

## Figures and Tables

**Table 1:** Eligibility Criteria (PICOS Framework).

Component	Description
(P)	Premenopausal or perimenopausal women, no restrictions on comorbidities. Studies involving men or mixed-gender populations without specific analysis for postmenopausal women were omitted.
(I)	The intervention group should receive beetroot-based interventions in any administration type, combined with any exercise modality.
(C)	Comparison groups included participants who received a placebo intervention—for example, nitrate-depleted beetroot juice or other inert substances—and engaged in an exercise training protocol of any type or intensity.
(O)	The primary outcomes assessed were cardiovascular parameters (including systolic and diastolic blood pressure, vascular function, and heart rate variability), besides measures of physical performance, such as peak oxygen uptake (VO_2peak_ or VO_2max_) and functional fitness test results.
(S)	Involved studies included single or double-blind, randomized controlled trials or randomized crossover-controlled designs. This study was restricted to articles published in peer-reviewed scientific research journals. Conference papers, master’s theses, doctoral dissertations, descriptive studies, case studies, editorials, and reviews were omitted.

**Table 2:** Description of the characteristics of the study population of articles by author and year, sample, age (years), intervention, control, outcomes and funding.

Author/ Years/Country	Study Design	Sample	Age (years)	Intervention	Control	Outcomes/Results	Limitations
Amaral et al. 2019/Brazil [19]	Double-blind, randomized, placebo-controlled, crossover trial.	13 hypertensive postmenopausal women.	58.1 ± 4.6 years.	Exercise Modality: Treadmill aerobic exercise. Intensity: 65–70% of heart rate reserve (HRR). Duration: 40 min. Beetroot Juice intervention: 35 mL of concentrated beetroot juice (Beet-It Sport Shot, Ashbocking, UK) diluted in 315 mL of distilled water with 6 g of non-caloric orange juice flavored powder (Clight, Brazil), totaling 350 mL of juice. Dose: 400 mg of nitrate (NO^3−^), equivalent to 20.78 mmol/kg of NO^3−^.	A nitrate-depleted version of the beetroot juice, prepared by filtering the BJ through an ion exchange resin to remove NO^3−^ (3.86 mmol/kg of NO^3−^).	There were no differences in post-exercise blood pressure reduction between intervention and placebo groups. No significant differences were detected between the three experimental conditions for systolic blood pressure, diastolic blood pressure, or heart rate (*p* = 1.000), and no correlation was found between salivary nitrite and blood pressure (SBP *p* = 0.749; DBP *p* = 0.618).	Blood flow was not directly assessed, which limits the mechanistic interpretation of the hemodynamic responses observed. Second, the intervention was acute, based on a single dose of beetroot juice, thereby restricting the generalizability of the findings to chronic supplementation scenarios. Moreover, the participants were under heterogeneous pharmacological treatment with different classes of antihypertensive drugs.
Amaral et al. 2021/Brazil [20]	Crossover, randomized, double-blind, placebo-controlled trial.	13 hypertensive postmenopausal women.	58.1 ± 4.6 years.	Exercise Modality: Treadmill aerobic exercise. Intensity: 65–70% of heart rate reserve (HRR). Duration: 40 min. Beetroot Juice intervention: 35 mL of concentrated beetroot juice (Beet-It Sport Shot, UK) diluted in 315 mL of distilled water with 6 g of non-caloric orange juice flavored powder (Clight, Brazil), totaling 350 mL of juice. Dose: 400 mg of nitrate (NO^3−^), equivalent to 20.78 mmol/kg of NO^3−^.	A nitrate-depleted version of the beetroot juice, prepared by filtering the BJ through an ion exchange resin to remove NO^3−^ (3.86 mmol/kg of NO^3−^).	There was a difference in decreasing catalase activity and GSH between the intervention and placebo groups. The catalase activity decreased after both high-nitrate and low-nitrate beetroot juice compared with the orange-flavored drink, with lower AUC values (*p* < 0.01). Salivary glutathione (GSH) also decreased only after the high-nitrate condition, with lower AUC compared to the control (*p* < 0.01). Time effects were observed for all variables: total protein increased significantly at 170′ compared to all other time points (*p* < 0.001); catalase was lower at 170′ than baseline (*p* < 0.001); GSH was higher at 130′ and 170′ versus baseline (*p* = 0.004); and FRAP was lower at 130′ and 260′ compared to baseline but higher at 170′ than 130′ (*p* < 0.001). No significant differences among conditions were found for FRAP responses (*p* = 0.680)	The participants were medicated hypertensive postmenopausal women, and the use of different antihypertensive drugs may have influenced the oxidative stress responses observed. Only the acute effects of beetroot juice ingestion in association with exercise were examined, without evaluation of chronic supplementation or the isolated impact of the beverages in the absence of exercise.
Benjamim et al. 2024/Brazil [21]	Randomized crossover, triple-blind, placebo-controlled trial.	14 postmenopausal women with systemic arterial hypertension (SAH).	59 ± 4 years.	Exercise Modality: Treadmill aerobic exercise. Intensity: 65–70% of the VO_2peak_. Duration: 30 min. Beetroot Juice Intervention: 70 mL of beetroot juice per bottle (Beet-It, Sports Shot, UL). Dose: 400 mg of nitrate (NO^3−^), equivalent to 6.4 mmol of NO^3−^.	A nitrate-depleted version of beetroot juice, prepared by filtering the BJ through an ion exchange resin to remove NO^3−^ (0.38 mmol of NO^3−^).	Acute Ingestion: Significant reduction in systolic blood pressure (SBP) post-exercise (−9.28 mmHg; *p* = 0.019). Improved flow-mediated dilation (FMD) post-exercise (3.18%; *p* = 0.031). Enhanced recovery of parasympathetic modulation (Heart Rate Variability indices improved). Short-term Ingestion (One Week): Increased FMD values both before (4.5%; *p* = 0.005) and after exercise (4.2%; *p* = 0.004). Faster recovery of parasympathetic modulation after exercise. No significant reduction in SBP or Diastolic Blood Pressure (DBP) compared to placebo after one week.	The small sample size of hypertensive postmenopausal women limits statistical power and generalizability to other populations, such as men, younger adults, or physically active individuals. The intervention assessed only acute and short-term (one week) supplementation, preventing conclusions about long-term effects or sustained adaptations.
Benjamim et al. 2024/Brazil [22]	Randomized, double-blind, placebo-controlled crossover trial.	15 postmenopausal women.	59 ± 4 years.	Exercise Modality: Physical performance tests. Intensity: Handgrip strength (peak force measured with three attempts); arm curl test (2.3 Kg weight for 30 s, recording the number of completed moves); sit-to-stand test (as many times as possible within 30 s); agility and dynamic balance test (best time recorded in seconds); 6 min walk test (the greatest distance possible in a perimeter of 45.7 m within 6 min time frame). Beetroot Juice Intervention: 70 mL of beetroot juice (Beet It, Sport Nitrate 400, UK). Dose: 400 mg of nitrate (NO^3−^).	70 mL of the nitrate-depleted version of beetroot juice.	The study suggests a slight improvement in the performance of the 6-min walk test (6MWT). NO^2−^ plasma concentrations were consistently elevated in the NO^3−^ condition at 0.41 (0.40) μM compared to the PLA at 0.18 (0.18) μM (*p* < 0.001). The 6MWT showed higher values in BRJ with NO^3−^ condition (19.6 m [95%CI: 1.33 to 37.88]; *p* = 0.038), while the other physical performance tests did not show a significant difference between conditions (*p* > 0.05).	The sample size was small (*n* = 14 completers), which reduces statistical power and limits generalizability beyond the specific cohort of postmenopausal women aged 50–65. The intervention duration was relatively short (8 days), thus precluding conclusions regarding longer-term effects or sustainability of benefits. Third, while one physical performance test (6 min walk test) showed improvement, the other performance outcomes (handgrip strength, arm curl, sit-to-stand, agility/dynamic balance) did not differ between conditions, indicating that benefits may be limited to tests of longer duration/cardiorespiratory demand.
Caldwell et al. 2019/USA [23]	2 d randomized, double-blind, placebo-controlled crossover.	10 postmenopausal women.	56 ± 1 years.	Exercise Modality: Isometric handgrip exercise. Intensity: 20% MVC. Duration: 7 min. Beetroot Juice intervention: 140 mL of concentrated NR (NO^3−^) beetroot juice supplement (Beet It Sport, James White Drinks Ltd., Ipswich, UK). Dose: 140 mL of concentrated NR (NO^3−^) containing 12.9 mmol NO^3−^.	140 mL of nitrate placebo beetroot juice supplement (PL; James White Drinks Ltd.) containing negligible (NO^3−^).	Resting SBP, DBP, HR, MAP, and SVR were not significantly different between treatments. During the SS exercise, forearm blood flow (FBF) and forearm vascular conductance (FVC) were lower in the nitrate condition compared to placebo. Plasma nitrite levels were significantly higher after the nitrate-rich (NR) supplement compared to the placebo (NP) condition (809 ± 146 vs. 79 ± 19 nM; *p* < 0.001). Resting systolic, diastolic, mean arterial pressure, and heart rate did not differ between treatments (*p* > 0.05). During steady-state handgrip exercise, forearm blood flow (NR: 190 ± 16 vs. NP: 218 ± 17 mL·min^−1^; *p* = 0.03) and forearm vascular conductance (NR: 159 ± 12 vs. NP: 177 ± 12 mL·min^−1^·100 mmHg^−1^; *p* = 0.03) were significantly lower in the NR condition.	Only an acute dose of dietary nitrate supplementation was tested; therefore, the potential benefits of chronic supplementation remain unknown. The sympathetic nerve activity was not measured, which limits the mechanistic interpretation of the observed improvements in functional sympatholysis.
Caldwell et al. 2024/USA [24]	Randomized, double- blind, crossover study design	Twelve postmenopausal females.	64 ± 5 years.	Exercise Modality: Isometric handgrip exercise. Intensity: ischemic exercise at 20% MVC and 20 contractions per min at 10, 15, and 20% MVC Duration: 3 min per stage and 3 min of ischemic exercise. Beetroot Juice intervention: nitrate-rich supplement in the form of beetroot juice. Dose: 140 mL of a concentrated beetroot juice supplement (Beet It Sport, James White Drinks Ltd., Ipswich, UK) containing [~12.9 mmol NO^3−^].	Nitrate-poor supplement in the form of black currant [<0.2 mmol NO^3−^], (Jungle Powders, Pärnumaa, Estonia).	Acute supplementation did not change resting or ischemic exercise FMD. Further, no changes were seen with resting or ischemic exercise PORH. Plasma nitrate and nitrite concentrations significantly increased following nitrate-rich beetroot juice compared with placebo (*p* < 0.01). Mean arterial pressure was reduced after supplementation, both at rest (*p* < 0.05) and during incremental small muscle mass exercise (*p* < 0.05).	The small sample size (*n* = 12 postmenopausal females), the intervention was acute, using a single dose of nitrate-rich beetroot juice, which precludes conclusions about effects with repeated or long-term supplementation.
Carrijo et al. 2021/Brazil [25]	Crossover, randomized, and double-blind study.	13 postmenopausal hypertensive women.	58.1 ± 4.6 years	Exercise Modality: Treadmill aerobic exercise. Intensity: 65–70% of HR reserve Duration: 40 min. Beetroot Juice intervention: high-NO^3−^ drink consisted of 35 mL concentrated beetroot juice diluted in 315 mL of distilled water with 6 g of non-caloric orange flavor powder (Clight, Mondelez International, Inc., São Paulo, Brazil), totaling 350 mL of juice. Dose: 400 mg of NO^3−^ (Beet-It Sport Shot, James White Drinks Ltd., Ipswich, United Kingdom), containing 20.78 mmol/kg of NO^3−^.	A non-caloric orange-flavored drink (OFD) made by diluting 6 g of orange-flavored powder in 350 mL of distilled water, with no nitrate content.	No differences were found between the HR average of the sessions. SDNN increased significantly post-exercise compared with baseline (*p* < 0.05), while RMSSD and PNN50 did not differ (*p* > 0.05). In the frequency domain, LF and LF/HF ratio increased after exercise (*p* < 0.05), whereas HF showed no significant changes (*p* > 0.05). In the non-linear domain, SD1, SD2, and SD2/SD1 all increased after exercise across conditions (*p* < 0.05). Importantly, there were no interaction effects between time and session (*p* > 0.05), indicating that beetroot juice—whether high- or low-nitrate—did not modify HRV responses when compared to the orange-flavored control drink	The sample size was small (*n* = 13). All participants were hypertensive postmenopausal women under pharmacological treatment, and the use of different classes of antihypertensive drugs may have masked the potential effects of nitrate supplementation on heart rate variability (HRV). Furthermore, the study examined only the acute effects of a single dose of beetroot juice, which restricts conclusions regarding chronic supplementation. In addition, HRV was assessed for 90 min after exercise, and longer recovery windows might provide complementary insights.
Hogwood et al. 2023/USA [26]	Randomized, double-blind, placebo-controlled trial	24 estrogen-deficient females postmenopausal	61 ± 5 (Placebo) 59 ± 5 Nitrate-rich beetroot juice	Exercise modality: Cycle ergometer exercise test. Visits consisted of vascular health measures before (time point 0) and every 30 min after (time points 60, 90, 120, 150, and 180) calorically matched high-intensity exercise (HIE), moderate-intensity exercise (MIE). Dose: 13 mmol NO^3−^ in the form of beetroot juice(BRJ; *n* = 12) or placebo (PL; *n* = 12) for 2 days before experimental visits and 2 h before testing.	The placebo was a nitrate-depleted beetroot juice with a similar appearance and taste to the nitrate-rich supplement.	Beetroot juice (BRJ) supplementation significantly increased plasma nitrate and nitrite and decreased serum endothelin-1 compared with placebo (all *p* < 0.001). Peak FMD improved with BRJ compared with placebo (*p* = 0.02), and exercise intensity also had a significant effect (*p* < 0.001), with a nonsignificant interaction (*p* = 0.11). Within-condition analyses showed that BRJ combined with high-intensity exercise (HIE) enhanced FMD compared with BRJ plus control (*p* = 0.05; *p* = 0.005 when baseline diameter was included as a covariate), while BRJ plus moderate-intensity exercise showed medium effect sizes but did not reach significance. Resting vascular measures, including blood pressure and pulse wave velocity, were not significantly affected by BRJ supplementation (*p* > 0.05), though systolic blood pressure decreased modestly after both placebo (*p* = 0.04) and BRJ (*p* = 0.01) supplementation	The relatively small sample size (*n* = 24, with 12 per treatment arm) was powered only for the primary outcome of peak flow-mediated dilation (FMD) and not for more complex interaction analyses. The majority of participants demonstrated relatively preserved vascular health, with only nine individuals showing impaired baseline FMD (<4.5%), potentially masking the magnitude of improvement achievable in less healthy cohorts.
Proctor et al. 2022/USA [27]	Double-blind, randomized crossover design	13 postmenopausal women (57–76 yr)	64 ± 1.4	Exercise modality: Physical performance test. Performed rhythmic isometric handgrip contractions (10% MVC, 30 per min) during progressive forearm blood flow restriction (upper arm cuff gradually inflated 20 mmHg each min). Dose:140 mL of NO^3−^ concentrated (9.7 mmol, 0.6 gm NO^3−^)	NO^3−^ depleted beetroot juice	These results suggest that acute NO^3−^ supplementation prolongs time-to-fatigue and speeds grip force development during progressive forearm muscle ischemia in postmenopausal women. Dietary nitrate supplementation significantly increased plasma nitrate and nitrite concentrations compared with placebo (*p* < 0.01). However, no significant differences were observed between conditions in flow-mediated dilation responses (*p* = 0.44) or resting blood pressure (systolic *p* = 0.21; diastolic *p* = 0.28). Heart rate also did not differ significantly between groups (*p* = 0.31). Subgroup analysis suggested that women with lower baseline FMD (<4.5%) tended to exhibit greater improvements with nitrate supplementation, but these changes did not reach statistical significance (*p* = 0.08).	The sample comprised only 13 postmenopausal women. The trial was short in duration, examining only acute supplementation and a 7-day intervention, which prevents conclusions about longer-term effects. Dietary intake beyond the supplementation protocol was not strictly controlled, and variability in background diet could have influenced nitrate bioavailability and vascular outcomes.
Zoughaib et al., 2023/USA [28]	Randomized, double-blind, placebo-controlled crossover design	Sixteen community-dwelling older men and women	71 ± 5 years old	First phase: Participants were divided into groups for daily supplementation with BRJ for two weeks, with or without NO. After this period, blood samples were collected. Second phase: Prior to physical activity, blood pressure was measured, and new blood samples were collected. Participants were instructed to perform an acute intake of BRJ containing 18.2 ± 6.2 mmol of NO^3−^, while another group received BRJ without NO. Participants first performed three maximal 5 s isometric contractions, with 15 s of rest between each repetition. After a 2 min rest, isokinetic testing was conducted, during which each participant performed three maximal knee extensions at each velocity, with 2 min of rest between each set. They then underwent physical exercise with muscle performance assessed via isokinetic dynamometry. After a 10 min recovery period, blood pressure was measured again, and additional blood samples were collected.	Participants were initially assigned to one of two groups: BRJ with NO^3−^ or BRJ without NO^3−^	Both acute and short-term supplementation with NO^3−^-rich BRJ have equally beneficial effects on muscle speed and power in older men and women. However, there were no changes in blood pressure or in plasma markers of oxidative stress Beetroot juice supplementation significantly elevated plasma nitrate and nitrite concentrations compared with the nitrate-depleted placebo (e.g., nitrite at 1 h, 2 h post-ingestion, and 10 min post-exercise; *p* = 0.0338, *p* = 0.0030, and *p* = 0.0030, respectively). Blood pressure outcomes (systolic, diastolic, and mean arterial pressure) showed only time (*p* = 0.0296) and duration (*p* = 0.0469) effects, with no treatment differences or interactions (all *p* ≥ 0.09).	The sample size was modest (*n* = 16 older men and women aged 65–79). Although increases in muscle speed and power were observed, the placebo/control (NO^3−^ depleted beetroot juice) also showed some practice-or learning effects, which complicates attribution of changes uniquely to nitrate. The duration (2 weeks) for short-term supplementation is still relatively short.

**Table 3:** Levels of evidence analysis via (GRADE Working Group, 2004).

Outcome	Number of Studies	Risk of Bias	Inconsistency	Indirectness	Imprecision	Certainty of Evidence
HF	2	Not serious	Very serious ^a^	Not serious	Very serious ^b^	Very low ⨁◯◯◯
RMSSD	2	Not serious	Very serious ^c^	Not serious	Not serious	Moderate ⨁⨁⨁◯
SDNN	2	Not serious	Very serious ^d^	Not serious	Serious ^e^	Low ⨁⨁◯◯

Explanations: ^a^ I^2^ = 86%; ^b^ High CI 95%; ^c^ I^2^ = 73%; ^d^ I^2^ = 81%; ^e^ High CI 95%—(RMSSD) Root Mean Square of Successive Differences; (HF) High Frequency (power of heart rate variability); SDNN Standard Deviation of Normal-to-Normal Intervals. ⨁◯◯◯: Very low certainty. ⨁⨁◯◯: Low certainty. ⨁⨁⨁◯: Moderate certainty.

## Data Availability

The data from this study are available online: https://drive.google.com/drive/folders/1AMyeMaXs-m8VxglI1maF6wG8wi3gZ86w (accessed on 22 September 2025).

## Abbreviations

The following abbreviations are used in this manuscript:

HRV	heart rate variability
VO_2_	Oxygen consumption
RMSSD	Root Mean Square of Successive Differences
HF	High Frequency (power of heart rate variability)
SDNN	Standard Deviation of Normal-to-Normal Intervals
NOS	nitric oxide synthases
NO	nitric oxide
PRISMA	Preferred Reporting Items for Systematic Reviews and Meta-Analyses
MD	Mean difference
BMI	Body Mass Index
OFD	orange-flavored control
IL-6	Interleukin-6

## Appendix A

**Table A1:** Search Strategy.

Database	Search Strategy
General Boolean Syntax	((“Beetroot supplementation” OR “beetroot juice” OR “beetroot extract” OR “dietary nitrate”) AND (“Postmenopausal women” OR “post-menopause” OR “menopause”) AND (“Exercise” OR “Physical Activity” OR “Strengthening Program” OR “Training” OR “Rehabilitation” OR “Habilitation” OR “heart rate variability”)
PUBMED	(((“Beetroot supplementation” [MeSH Terms] OR “beetroot juice” OR “beetroot extract” OR “dietary nitrate” [MeSH Terms]) AND (“Postmenopausal women” [MeSH Terms] OR “post-menopause” OR “menopause” [MeSH Terms]) AND (“Exercise” [MeSH Terms] OR “Physical Activity” [MeSH Terms] OR “Strengthening Program” OR “Training” [MeSH Terms] OR “Rehabilitation” [MeSH Terms] OR “Habilitation”) OR “heart rate variability”))
EMBASE	((‘beetroot supplementation’ OR ‘beetroot juice’ OR ‘beetroot extract’ OR ‘dietary nitrate’) AND (‘postmenopausal women’ OR ‘post-menopause’ OR ‘menopause’) AND (‘exercise’ OR ‘physical activity’ OR ‘strengthening program’ OR ‘training’ OR ‘rehabilitation’ OR ‘habilitation OR “heart rate variability”))
LILACS	(“beetroot supplementation” OR “beetroot juice” OR “beetroot extract” OR “dietary nitrate”) AND (“postmenopausal women” OR “post-menopause” OR “menopause”) AND (“exercise” OR “physical activity” OR “strengthening program” OR “training” OR “rehabilitation” OR “habilitation” OR “heart rate variability”)
Cochrane Library	(“Beetroot supplementation” OR “beetroot juice” OR “beetroot extract” OR “dietary nitrate”) AND (“Postmenopausal women” OR “post-menopause” OR “menopause”) AND (“Exercise” OR “Physical Activity” OR “Strengthening Program” OR “Training” OR “Rehabilitation” OR “Habilitation”)
SCOPUS	((“beetroot supplementation” OR “beetroot juice” OR “beetroot extract” OR “dietary nitrate”) AND (“postmenopausal women” OR “post-menopause” OR “menopause”) AND (“exercise” OR “physical activity” OR “strengthening program” OR “training” OR “rehabilitation” OR “habilitation” OR “heart rate variability”)
Web of Science	(“beetroot supplementation” OR “beetroot juice” OR “beetroot extract” OR “dietary nitrate”)AND (“postmenopausal women” OR “post-menopause” OR “menopause”) AND (“exercise” OR “physical activity” OR “strengthening program” OR “training” OR “rehabilitation” OR “habilitation” OR “heart rate variability”)
CINAHL	((MH “Beetroot supplementation” OR “beetroot juice” OR “beetroot extract” OR “dietary nitrate”) AND (MH “Postmenopausal Women” OR “post-menopause” OR “menopause”) AND (MH “Exercise” OR “Physical Activity” OR “Strengthening Program” OR “Training” OR “Rehabilitation” OR “Habilitation”))
